# Beyond the Smile: Exploring the Mental Well‐Being of Dental Students Across Institutions

**DOI:** 10.1111/eje.13085

**Published:** 2025-02-24

**Authors:** Kamran Ali, Daniel Zahra, Ulfat Bashir, Hina Zafar Raja, Usman Sana, Asma Shakoor, Mariya Khalid, Amna Mansoor, Mahwish Raja

**Affiliations:** ^1^ QU Health College of Dental Medicine Qatar University Doha Qatar; ^2^ School of Psychology Plymouth University Plymouth UK; ^3^ Islamic International Dental College Riphah International University Islamabad Pakistan; ^4^ CMH Institute of Dentistry Lahore Pakistan; ^5^ Azra Naheed Dental College Superior University Lahore Pakistan; ^6^ Sardar Begum Dental College Gandhara University Peshawar Pakistan; ^7^ KMU Institute of Dentistry Kohat Pakistan

**Keywords:** dental students, mental health, risk factors, student support, universities

## Abstract

**Introduction:**

A high frequency of mental issues has been reported amongst dental students in recent years. The aim of this study was to explore the frequency of depression, stress, and anxiety amongst undergraduate dental students in a developing country and identify factors which may contribute to the poor mental health of dental students.

**Materials and Methods:**

After obtaining ethical approval, undergraduate dental students from 14 dental institutions were invited to participate in an online study. Data were collected using two globally validated scales for screening mental health. The survey inventory also included two open‐ended items and was administered using Google forms.

**Results:**

Complete responses were received from 639 participants, which included 71.67% (*n* = 458) females and 28.33% (*n* = 181) males. The overall response rate was 43%. The modal age group was 18–21‐year‐olds (63.54%, *n* = 406), followed by 22–25‐year‐olds (35.52%, *n* = 227). The mean score on PHQ‐9 was 10.37 (SD ± 6.13) and 48.67% of participants showed moderate to severe depression. The mean DASS–21 score was 20.81 (SD ± 14.64) and 48.21% of participants were screened positively for moderate to extremely severe depression, 49.30% for moderate to extremely severe anxiety, and 30.36% of participants showed features of moderate to extremely severe stress. Significantly positive correlations were observed for the whole sample and demographic factors for participant scores on PHQ‐9 for Depression, and Depression, Anxiety, and Stress scores on DASS‐21. Academic workload, social interactions, personal factors, academic environment, and financial difficulties were reported as the main causes of poor mental health.

**Discussion:**

This study shows a high prevalence of depression, anxiety, and stress amongst undergraduate dental students in a country with a unique socio‐cultural landscape. The study also identified underlying factors which adversely affect the mental health of dental students and provides recommendations to address these challenges.

## 1 Introduction

Mental health issues are being reported increasingly amongst students in medicine, dentistry, nursing, and other allied healthcare professions [1, 2, 3, 4, 5]. Although the demanding nature of healthcare education and practice has been associated with increased risk of psychological stress for over a century, mental health issues amongst healthcare students are being reported with increasing frequency, especially following the COVID‐19 pandemic [6, 7, 8, 9, 10, 11, 12]. Risk of infection, restrictions to social mobility, and interruption of clinical training during the COVID‐19 pandemic worsened social isolation and created uncertainty about the future. The students were required to adapt quickly to remote learning and assessments, which added to their stress and anxiety [13].

Mental health issues may be precipitated by a multitude of factors such as academic and clinical workload, fear of failure, social and relational issues, financial constraints, and inadequate institutional support [2, 5, 14]. Poor mental health may also be associated with impostor syndrome and negative feelings (inadequacy, shame, and embarrassment) [15]. Psychological problems may manifest as burnout, anxiety, stress, and depression and can, in turn, lead to adverse effects on physical health [16]. Disorders related to eating, sleep, addiction, and suicidal tendencies are also reported amongst healthcare students experiencing mental health issues [11, 12, 17, 18, 19, 20]. The implications of poor mental health of healthcare students extend well beyond the temporal confines of university settings. Healthcare students represent the future workforce, and poor mental health can result in prolonged or frequent absence from work and may, in worse cases, force healthcare professionals to leave the profession, resulting in significant adverse impacts on the healthcare workforce and services to the community [18, 21, 22, 23].

A significant body of literature has been published on mental health conditions amongst medical and dental students in developed countries. However, less is known about the scale of mental health issues and underlying risk factors affecting dental students in developing countries. Pakistan ranks as the fifth most populous nation globally, and the rapid population growth has adversely affected human development and quality of life [24, 25]. Currently, there are 61 dental institutions in the country, including 18 in the public sector and 48 in the private sector [26]. The majority of the dental institutions are located in Punjab (*n* = 30) including seven institutions in the federal capital Islamabad, followed by Sindh (*n* = 19) and KPK (*n* = 11) while Baluchistan only has one dental institution (*n* = 1). All dental institutions in Pakistan offer a 4‐year Bachelor of Dental Surgery (BDS) programme. The economy of Pakistan is unstable, with a rising foreign debt and limited investment in public infrastructure. Consequently, dental graduates in Pakistan have limited employment opportunities in the public sector, and the majority lack financial resources to set up a private dental practice. In addition, dental students in Pakistan do not have access to structured career counselling, which may lead to uncertainty about their future career pathway. These challenges may have a negative impact on the mental health of dental students and new graduates [27].

The aim of this study was to explore the frequency of depression, stress, and anxiety amongst undergraduate dental students in a developing country and identify factors which may contribute to the poor mental health of dental students.

## 2 Methods

### 2.1 Research Ethics

Research ethics approval for this study was obtained from the institutional ethics review committee (Approval Number: ANMC/ERB/23 dated 16/02/2023). Participation in the study was voluntary, and all data were collected and processed anonymously. All participating students provided informed consent before providing their responses to the survey.

### 2.2 Study Design

This research is an analytical cross‐sectional study based on an online survey using Google forms.

### 2.3 Sample Size Calculation

Power analysis with G*Power software (version 3.1) was used to calculate the sample size for this study [28]. The required sample size parameters were estimated to be between 386 and 546 with 8°–12° of freedom, *α* = 0.05, and a power of 0.90 to detect small‐to‐medium effects (*w* = 0.2). These parameters were also appropriate to detect small effect sizes using a *t*‐test to compare differences in mean scores between independent groups.

### 2.4 Sampling Technique and Participants

A non‐randomised probability sampling technique was used to recruit undergraduate dental students at 14 dental institutions across four regions (Punjab, KPK, Sindh, and Baluchistan) of the country. Students who had experienced interruptions in their studies were not included. The target participants were approached through the Dean/Head of each institution and invited to participate in an online survey. The administrator at each institution also acted as a gatekeeper for sending the invitations to the students. A reminder was sent after 3 weeks.

### 2.5 Data Collection

The survey inventory was divided into five sections and was administered online using Google forms (The full questionnaire is attached as an Appendix S1).

The first section required the participants to provide informed consent confirming that their participation was voluntary and that they understood the purpose and scope of the study and that all data related to the study would be processed anonymously. The second section related to demographic information of the participants including age, gender, institution, year of study, and financial support. The third section was based on a nine‐item version of the patient health questionnaire (PHQ‐9) which is a validated and widely used instrument used to screen for depression [29]. The fourth section included a 21‐item Depression, Anxiety and Stress Scale (DASS21). DASS‐21 is also a validated and widely used instrument to screen for mental health issues [30]. Each of the three subscales within DASS‐21 consists of 7 items scored using a Likert scale ranging from 0 to 3. The scoring structure and cut‐off points for PHQ‐9 and subscales are elaborated further in the results section. The last section consisted of two open‐ended items. The first item related to perceived factors which may be responsible for adverse effects on participants' mental health. The second item asked participants to provide recommendations for improving support for students with poor mental health.

### 2.6 Data Analyses

Data analyses were conducted using the R statistical environment for Windows (R Core Team, 2022) [31]. Descriptive statistics were used to evaluate the distributions of scores for each scale for the entire sample and subgroups. Chi‐squared tests of association were conducted to compare the severity of symptoms between groups on each scale. Correlation coefficients were calculated to assess the relationships between scores of participants.

## 3 Results

### 3.1 Demographic Profile

Of the 705 responses received, 640 provided complete PHQ‐9 and DASS‐21 scales, and these form the basis of the analyses; participants with any missing PHQ‐9 or DASS‐21 item responses were excluded as thresholds are based on summation across items, and scaling scores to account for missing data would complicate the interpretation of the findings. One participant was excluded from the subsequent analyses to safeguard anonymity. Of these 639 remaining complete responses, 71.67% (*n* = 458) were from female participants, 28.33% (*n* = 181) from male participants. The overall response rate was 42%.

The modal age group was 18–21‐year‐olds (63.54%, *n* = 406), followed by 22–25‐year‐olds (35.52%, *n* = 227), with a minority being older (0.63%, *n* = 4, at 26–29; 0.31%, *n* = 2 at 30 or more). The majority of age‐based analyses focus on the comparison of the 18–21‐ and 22–25‐year‐old age groups. All stages of study were represented; Year 1, 31.92%, *n* = 204; Year 2, 20.81%, *n* = 133; Year 3, 14.55%, *n* = 93; and Year 4, 32.71%, *n* = 209.

The geographic location of the participants, financial support, and institution type are shown in Table 1.

**TABLE 1:** Institution characteristics.

Region			Financial support			Institution type		
	*n*	%		*n*	%		*n*	%
Punjab	298	46.64	Self‐finance	509	79.66	Private	572	89.51
Islamabad	185	28.95	Other	53	8.29	Public	59	9.23
KPK	113	17.68	Sponsorship	44	6.89	Other	8	1.25
Sindh	31	4.85	Scholarship	32	5.01			
Baluchistan	12	1.88	Not specified	1	0.16			

### 3.2 Scoring Structure

Responses to PHQ‐9 were scored from 0 to 3 for each item as follows: Not at all = 0, Several days = 1, More than half the days = 2, and Nearly every day = 3. The total PHQ‐9 score was calculated by summing the scores across all nine items, and the resulting score was classified into depression severity categories: None (0–4), Mild (5–9), Moderate (10–14), Moderately Severe (15–19), and Severe (20–27).

Responses to DASS‐21 were scored from 0 to 2 for each item as follows: Did not apply to me at all = 0, Applied to me to some degree, or some of the time = 1, Applied to me to a considerable degree or a good part of the time = 2, Applied to me very much or most of the time = 3. Item scores were then summed to give an overall DASS‐21 score. Subscale scores for relevant items were summed to yield scores for Depression (Items 3, 5, 10, 13, 16, 17, 21), Anxiety (Items 2, 4, 7, 9, 15, 19, 20), and Stress (Items 1, 6, 8, 11, 12, 14, 18). These subscale scores were then used to categorise the severity of depression, anxiety, and stress using standard thresholds of the scale as summarised in Table 2.

**TABLE 2:** DASS‐21 Thresholds for subscale severity categories.

Severity category	DASS‐21 subscale		
	Depression	Anxiety	Stress
Normal	0–4	0–3	0–7
Mild	5–6	4–5	8–9
Moderate	7–10	6–7	10–12
Severe	11–13	8–9	13–16
Extremely severe	14+	10+	17+

### 3.3 Descriptive Statistics

Descriptive statistics for each scale score and the numbers of respondents in each category are shown in Tables 3 and 4.

**TABLE 3:** PHQ‐9 and DASS‐21 descriptive statistics by scale and subscale.

Statistic	PHQ‐9	DASS‐21			
	Total	Total	Depression	Anxiety	Stress
Mean	10.37	20.81	7.07	6.32	7.41
SD	6.13	14.64	5.55	4.99	5.09
Min	0	0	0	0	0
Max	27	63	21	21	21
Range	27	63	21	21	21
IQR	8	20	9	7	7

**TABLE 4:** Categorisation by scale and subscale.

Category	Frequency				Percentage of respondents			
	PHQ9	DASS‐21			PHQ9	DASS‐21		
		Dep.	Anxiety	Stress		Dep.	Anxiety	Stress
None	112	—	—	—	17.53	—	—	—
Normal	—	249	231	362	—	38.97	36.15	56.65
Mild	216	82	93	83	33.80	12.83	14.55	12.99
Moderate	159	146	92	89	24.88	22.85	14.40	13.93
Moderately severe	89	—	—	—	13.93	—	—	—
Severe	63	67	72	62	9.86	10.49	11.27	9.70
Extremely severe	—	95	151	43	—	14.87	23.63	6.73

Significantly positive correlations were observed for PHQ‐9 depression scores and DASS‐21 depression, anxiety, and stress scores for the whole sample, and within each subgroup of each factor (within males, females, 18–21‐year‐olds, 22–25‐year‐olds, private, public, and other institution types, self‐ and participants with ‘other’ financial support). All correlations were statistically significant at the *p* < 0.001 level. The correlation coefficients and *p* values for the entire sample are shown in Table 5.

**TABLE 5:** Pearson correlation coefficients (r) for correlations between scales.

	DASS‐21		
	Depression	Anxiety	Stress
PHQ‐9 total	0.72 (*p* < 0.001)	0.63 (*p* < 0.001)	0.70 (*p* < 0.001)
DASS21			
Depression	—	0.78 (*p* < 0.001)	0.83 (*p* < 0.001)
Anxiety	—	—	0.84 (*p* < 0.001)

#### 3.3.1 PHQ‐9

##### 3.3.1.1 Gender

Significantly higher scores on PHQ‐9 were observed for female respondents (M = 10.73, SD = 6.23) than for male respondents (M = 9.46, SD = 5.80; *t*(352.36) = 2.44, *p* = 0.015). However, the severity of depression categorisations between females and males was comparable; *χ* ^2^(4) = 7.03, *p* = 0.134.

##### 3.3.1.2 Year of Study

Year of Study was found to have an overall main effect on PHQ‐9 scores; *F*(3,635) = 6.63, *p* < 0.001, with Tukey HSD pairwise comparisons showing this to be underpinned by statistically significant differences between PHQ‐9 scores of those in Year 4 as compared to Year 1 (*p* < 0.001), Year 2 (*p* = 0.005), and Year 3 (*p* = 0.004). These differences are also reflected in an association between Year of Study and PHQ‐9 depression severity category, *χ* ^2^(12) = 29.05, *p* = 0.004.

##### 3.3.1.3 Financial Support

The financial support status of respondents, when considered in terms of Self‐Financed vs. Other (Sponsorship, Scholarship, some other form financial support status, or not specified) showed no association with PHQ‐9 depression severity category; *χ* ^2^(4) = 3.11, *p* = 0.540.

##### 3.3.1.4 Institution Type

No association was observed for Institution Type (Public, Private, or Other) with PHQ‐9 depression severity category; *χ* ^2^(8) = 4.38, *p* = 0.838.

##### 3.3.1.5 Age Group

Age Group was associated with PHQ‐9 depression severity categories, with a larger proportion of ‘Mild’ categorisations in the 18–21‐year‐old group than the 22–25‐year‐old group, and a larger proportion of ‘Severe’ categorisations in the 22–25‐year‐old group; *χ* ^2^(4) = 12.99, *p* = 0.011 (Table 6). Other Age Groups were excluded from this analysis due to small numbers in each category.

**TABLE 6:** PHQ‐9 Depression severity category by age group.

Age group	PHQ‐9 depression severity category					
	None	Mild	Moderate	Moderately severe	Severe	Total
18–21						
*N*	81	145	91	58	31	406
%_group_	19.95	35.71	22.41	14.29	7.64	100
22–25						
*N*	31	69	65	30	32	227
%_group_	13.66	30.40	28.63	13.22	14.10	100

#### 3.3.2 DASS‐21

##### 3.3.2.1 Gender

Males and females showed comparable proportions in each subscale severity category of DASS‐21: Depression (*χ* ^2^(4) = 3.76, *p* = 0.440), Anxiety (*χ* ^2^(4) = 4.87, *p* = 0.301), and Stress (*χ* ^2^(4) = 7.62, *p* = 0.107).

##### 3.3.2.2 Year of Study

Year of Study was associated with DASS‐21 Depression (*χ* ^2^(12) = 24.00, *p* = 0.020) and Stress severity (*χ* ^2^(12) = 41.88, *p* < 0.001), but not Anxiety severity (*χ* ^2^(12) = 17.10, *p* = 0.146) as shown in Table 7.

**TABLE 7:** DASS‐21 subscale severity by year of study.

Year of study	Frequency					Percentage (of Year)				
	Normal	Mild	Mod.	Severe	Extremely severe	Normal	Mild	Mod.	Severe	Extremely severe
Depression										
1	94	24	47	17	22	46.08	11.76	23.04	8.33	10.78
2	55	19	26	14	19	41.35	14.29	19.55	10.53	14.29
3	41	11	24	6	11	44.09	11.83	25.81	6.45	11.83
4	59	28	49	30	43	28.23	13.40	23.44	14.35	20.57
Anxiety										
1	91	29	22	20	42	44.61	14.22	10.78	9.80	20.59
2	48	17	21	17	30	36.09	12.78	15.79	12.78	22.56
3	35	12	17	8	21	37.63	12.90	18.28	8.60	22.58
4	57	35	32	27	58	27.27	16.75	15.31	12.92	27.75
Stress										
1	140	17	21	21	5	68.63	8.33	10.29	10.29	2.45
2	76	15	25	12	5	57.14	11.28	18.80	9.02	3.76
3	53	16	9	8	7	56.99	17.20	9.68	8.60	7.53
4	93	35	34	21	26	44.50	16.75	16.27	10.05	12.44

##### 3.3.2.3 Financial Support

The financial support status of respondents, when considered in terms of Self‐Financed vs. Other (Sponsorship, Scholarship, some other form financial support status, or not specified) showed no association with DASS‐21 Depression (*χ* ^2^(4) = 5.33, *p* = 0.256), Anxiety (*χ* ^2^(4) = 0.61, *p* = 0.961), or Stress (*χ* ^2^(4) = 2.44, *p* = 0.655) severity categories.

##### 3.3.2.4 Institution Type

No association was observed for Institution Type (Public, Private, or Other) with DASS‐21 Depression (*χ* ^2^(8) = 4.16, *p* = 0.855), Anxiety (*χ* ^2^(8) = 3.29, *p* = 0.930), or Stress (*χ* ^2^(8) = 7.18, *p* = 0.504) severity categories.

##### 3.3.2.5 Age Group

**FIGURE 1:**
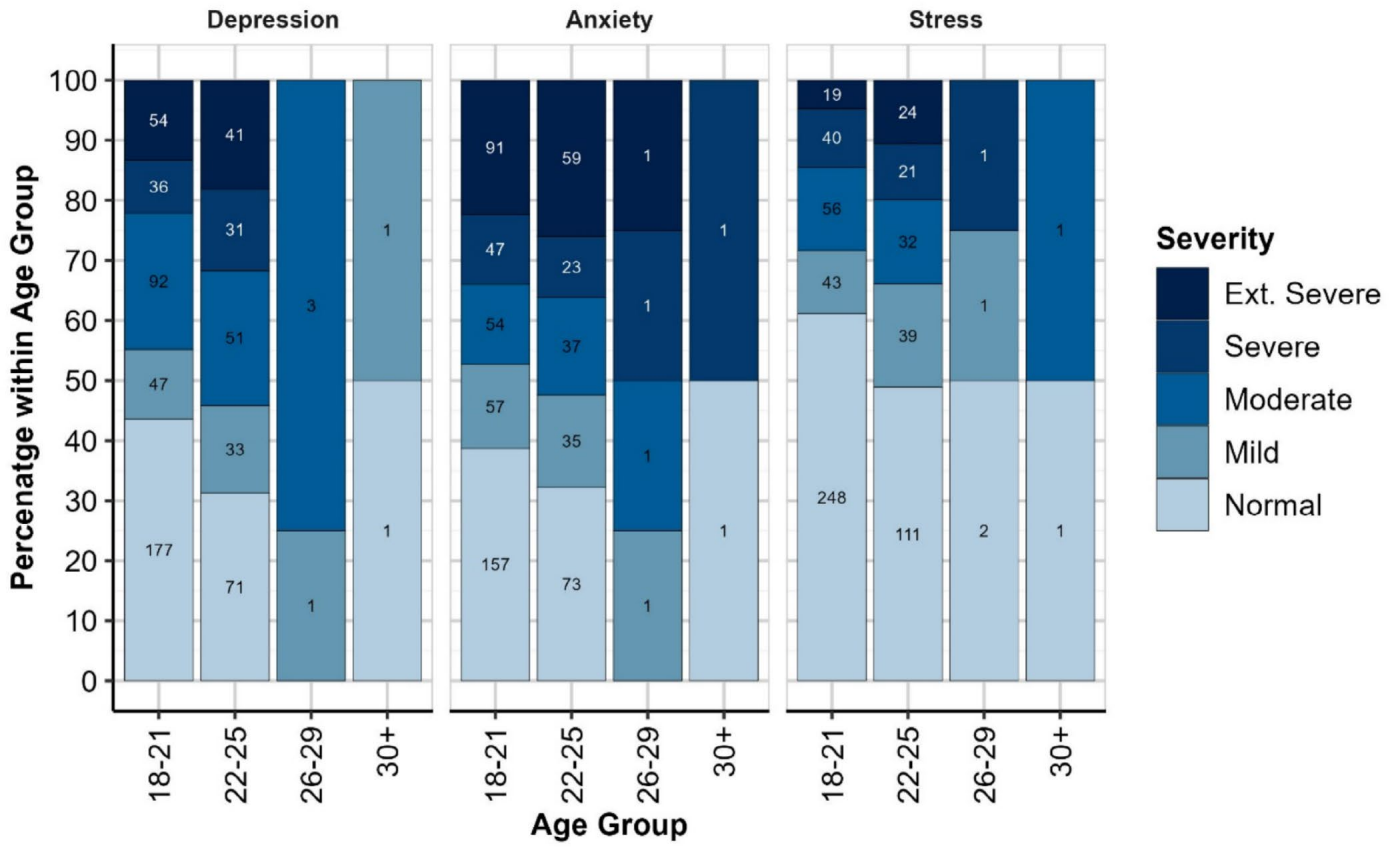
DASS‐21 Subscale severity by age group. Values within each bar indicate the frequency of respondents in that category.

Age Group was associated with DASS‐21 Depression (*χ* ^2^(4) = 12.01, *p* = 0.017) and Stress severity (*χ* ^2^(4) = 16.20, *p* = 0.003), but not Anxiety severity categories (*χ* ^2^(4) = 3.86, *p* = 0.425) as depicted in Figure 1. As with PHQ‐9 Age Group analyses, the 26–29 and 30+ Age Groups have been excluded from these analyses due to small numbers in each category.

### 3.4 Responses to Open‐Ended Questions

A thematic analysis was undertaken to analyse responses to open‐ended questions. Underlying factors perceived to affect mental health were reported by 602 participants and are summarised in Table 8. Workload related to academic activities and assessments, along with fear of failure in exams, were reported as a major factor. The participants also highlighted the need to improve transparency and fairness in the assessments, as some participants felt that the criteria of assessments and expected levels of student performance were not shared with the students consistently. Secondly, social interactions were reported to be a source of distress, as some participants felt socially isolated. Experiences on social media were also perceived to be a negative influence. Issues related to stress management and lack of time to address personal needs were also a strong theme. Finally, lack of support by the faculty, uncertainties related to future career prospects, and financial difficulties were highlighted as additional challenges for maintaining mental health and well‐being.

**TABLE 8:** Factors responsible for adverse effects on the mental health of participants.

Theme	Sub‐themes	Frequency
Academic workload	Burn‐out due to endless lectures and assignments Limited time to focus on self‐directed learning and personal development Assessment overload Fear of failure in exams	++++
II Social interactions	Isolation and loneliness Lack of peer support Unrealistic expectations from parents Negative influence of social media: bullying, judgemental behaviours, interference with sleep, time wastage	+++
III Personal factors	Lack of coping mechanisms: overthinking, negative self‐talk, poor stress management, imposter syndrome Previous psychological trauma Limited time to focus on physical exercise, spiritual growth and meet religious obligations	+++
IV Uncertainty about future	Limited career opportunities in the current job market Poor political and financial climate in the country	+++
V Academic environment	Faculty staff lack understanding and empathy Lack of equal opportunities and discrimination Lack of support for students experiencing mental health issues Inadequate career counselling	++
VI Financial pressures	High tuition fee	++

Lastly, the participants provided recommendations to improve the support for students with poor mental health. The main recommendations are summarised in Table 9. The responses underscored the need to moderate the workload related to academic activities and assessments and improve quality assurance mechanisms to oversee teaching, learning, and assessment practices with input from student representatives. The participants also highlighted the need to establish a dedicated mental health support system at their institution along with faculty training and the use of peer support groups. Finally, the participants recommended improved opportunities for recreational and social activities to counter stress related to academic activities.

**TABLE 9:** Recommendations to support students with poor mental health.

Theme	Sub‐themes	Frequency
Moderate academic workload	Solicit student representation in curriculum development and planning of learning activities Remove redundant and repetitive learning content Provide more time to students for self‐directed learning Improve quality assurance of teaching and assessment	++++
II Develop a mental health support system	Develop dedicated support for mental health of students Organise events to raise awareness about mental health Provide training to teaching faculty to identify and support students and refer students with poor mental health for counselling Develop peer support groups	++++
III Improve institutional environment	Provide opportunities and space for sports and recreational activities Organise social gatherings for the students	++++

## 4 Discussion

This is the first large‐scale multi‐institutional study on the mental health of undergraduate dental students in Pakistan to the best of the authors' knowledge. Undergraduate dental students representing 22.95% of dental institutions across all regions of the country participated in the study. The study provides useful insights into the scale of mental health problems and also identifies the underlying factors which adversely affect the mental health of the dental students in a developing country. Although the response rate was moderate, the sample size was well above the estimated power calculations, and undergraduate dental students from institutions across the country participated in the study. The study utilised PHQ‐9 and DASS‐21, which are two globally recognised instruments to evaluate depression, stress, and anxiety, and demonstrate good validity and reliability [32, 33, 34, 35]. The results of the current study showed strong positive correlations between PHQ‐9 and DASS‐21 scores, which provide further evidence of the reliability of these scales when used simultaneously.

The results of the current study show mild–moderate levels of depression, anxiety, and stress were identified in over 50% of the participants, and severe depression, anxiety, and stress were flagged up in approximately 10% of the participants. The scores indicate a high frequency of mental health issues amongst the participants and are broadly comparable to those reported amongst medical and dental students from other parts of the globe [35, 36, 37, 38, 39]. A recent study on dental students in Brazil reported that depression, anxiety, and stress were observed in 60.0%, 59.8%, and 60.9% of participants respectively [40]. A higher frequency of depressive symptoms was observed in a large‐scale study involving 21 dental schools in the United States of America, with 33.6% of participants reporting symptoms of mild depression, and 42.3% reporting symptoms suggestive of moderate, moderately severe, or severe depression [41]. Finally, a systematic review published in 2024 pooled data from 45 studies on mental health amongst dental students. The reported prevalence of depression was 38% (95% CI: 32%–44%); anxiety 48% (95% CI: 41%–55%) and sleep disorders 31% [95% CI: 24%–38%] [42]. These findings show a high prevalence of mental health issues amongst dental students and underscore the need to address mental health issues in undergraduate dental education programmes.

Although mental health problems amongst university students are reported widely in the literature, the socio‐cultural environment in Pakistan presents additional barriers for students with poor mental health. Lack of awareness regarding mental health, limited access to professional medical services, risk of social stigma, and resistance by parents and extended family members often prevent students from seeking appropriate professional help [43]. Moreover, a widespread network of self‐acclaimed faith healers exists in the country, and some people may end up falling victim to such scams, resulting in inappropriate treatments and worsening of their mental health issues [44]. Although the universities are recognising the need for mental health support, a structured support system is not available in most universities, and students facing mental health issues often end up suffering in silence unless they have the resources to seek professional help outside the university. Lack of institutional support leads to the deterioration of academic performance, withdrawal from higher education programmes, and also increases the risk of substance abuse and suicide [45, 46, 47]. Students may also avoid seeking help due to concerns regarding confidentiality and potential negative impact on their professional career [48].

The stigma associated with mental health conditions is reported widely and warrants a change in attitudes towards students and faculty experiencing poor mental health [49, 50, 51, 52]. The results of the current study show there is a need to raise awareness about mental health and in particular provide training to teaching faculty to support students experiencing mental health issues. It is also important that the relevant government authorities make it mandatory for all universities to establish a mental health support system for students to provide assistance, screening, counselling, and referral as needed. Faculty training to support students with poor mental health is a fundamental component of student support, and faculty training is now provided routinely in universities in developed countries [53, 54]. However, such practices are non‐existent in Pakistan and many other developing countries, and appropriate steps are warranted to address this gap.

Participants also reported financial difficulties as a factor for their poor mental health. A systematic review and meta‐analysis has also identified financial constraints as a key risk factor for mental health problems amongst university students [55]. Although financial stressors are also reported amongst medical students from high‐income countries, Pakistan is currently going through a phase of political turmoil and economic downturn with unprecedented inflation and growing joblessness [56]. The university fee for private dental students is particularly high, and given that a significant proportion of the participants in this study were from private institutions, it is understandable that financial difficulties were reported to have an adverse effect on the mental health of the participants. The poor economic climate of the country also generated feelings of uncertainty and job insecurity amongst the participants in the current study. Recent studies have shown a growing trend of brain drain from Pakistan, and given an opportunity, up to 66% of medical students and medical professionals would prefer to emigrate to a Western country in search of a better standard of living and to build their professional career [57, 58].

The key challenges reported by the participants were academic workload, performance in assessments, and risk of failure. These factors are well recognised in the literature and may have a negative impact on the mental health of students in healthcare professions [1, 4, 8, 9, 12, 18, 59]. Dental educators should streamline teaching, learning, and assessments to moderate the workload for the students. The focus should be on empowering the students to become independent and lifelong learners with skills in clinical problem‐solving, effective communication, team working, and professionalism [60, 61]. Dental educators in Pakistan need to work closely with the students and treat them as partners rather than mere recipients of education. Student representation on institutional committees responsible for curriculum review and assessments is recommended to address students' perspectives and concerns [62].

A negative impact of social media on mental health was reported by some participants in this study. Social media is a double‐edged sword, and while it offers numerous opportunities for connectivity and networking, there are growing concerns regarding its negative impact on mental health, especially amongst young people [63]. A recent umbrella review encompassing multiple systematic reviews and meta‐analyses shows inconsistent results regarding the association of mental health problems with social media use amongst young people [64]. Social media use is widespread and may precipitate negative feelings for some people; responsible use for professional networking and education may actually be beneficial [65]. Therefore, instead of treating social media as a stressor, institutions can provide students training on responsible and effective use of social media.

Some limitations of the current study need to be acknowledged. A combination of closed‐ended items on the PHQ‐9 and DASS‐21 scales and open‐ended items was used for data collection. A deeper understanding of the experiences and perceptions of students experiencing mental health issues could have been achieved with qualitative methods such as in‐depth interviews and focus groups. Moreover, the underlying factors that contribute to poor mental health could be explored more comprehensively with qualitative methods. Nevertheless, this study provides useful insights into the scale of mental health issues amongst undergraduate dental students in a developing country with a unique socio‐cultural landscape. The study also identified some of the underlying factors that adversely affect the mental health of dental students and provides recommendations to address these challenges.

## 5 Conclusion

This study assessed the mental health of undergraduate dental students across 14 dental institutions in Pakistan, and identified significant challenges. Over 60% of students experienced varying degrees of depression and anxiety, while approximately 43% exhibited signs of stress. Key contributing factors included academic workload, limited social interactions, personal challenges, and uncertainty about future career prospects as a dentist. These findings reiterate an urgent need for structured institutional initiatives to support students struggling with mental health issues and promote their overall well‐being.

## Author Contributions

Kamran Ali conceptualised the study, developed the methodology, and drafted the manuscript. Daniel Zahra conducted the data analyses. Ulfat Bashir, Hina Zafar Raja, Asma Shakoor, Mariya Khalid, and Amna Mansoor contributed to data collection. Usman Sana contributed to the ethics application and data collection. Mahwish Raja contributed to methodology and initial drafting of the manuscript. All authors reviewed and approved the manuscript.

## Conflicts of Interest

The authors declare no conflicts of interest.

## Supporting information


Appendix S1

## Data Availability

The data underlying this article will be shared on reasonable request to the corresponding author.

## References

[1] T. C. Wilkes , T. Lewis , M. Paget , et al., “Wellbeing and Mental Health Amongst Medical Students in Canada,” International Journal of Social Psychiatry 68 (2021): 1283–1288, 10.1177/00207640211057724. 34791951 PMC9465500

[2] J. Galvin , E. Suominen , C. Morgan , E. J. O'Connell , and A. P. Smith , “Mental Health Nursing Students' Experiences of Stress During Training: A Thematic Analysis of Qualitative Interviews,” Journal of Psychiatric and Mental Health Nursing 22, no. 10 (2015): 773–783, 10.1111/jpm.12273. 26459938

[3] K. M. Graner , A. B. A. De Moraes , A. R. Torres , M. C. P. Lima , G. S. Rolim , and A. T. De Abreu Ramos‐Cerqueira , “Prevalence and Correlates of Common Mental Disorders Among Dental Students in Brazil,” PLoS One 13, no. 9 (2018): e0204558, 10.1371/journal.pone.0204558. 30261025 PMC6160106

[4] T. Maragha , L. Donnelly , C. Schuetz , H. C. von Bergmann , and M. Brondani , “Mental Health and Wellness in Canadian Dental Schools: Findings From a National Study,” Journal of Dental Education 86, no. 1 (2022): 68–76, 10.1002/jdd.12768. 34402063

[5] A. Uraz , Y. S. Tocak , C. Yozgatlıgil , S. Cetiner , and B. Bal , “Psychological Well‐Being, Health, and Stress Sources in Turkish Dental Students,” Journal of Dental Education 77, no. 10 (2013): 1345–1355, 10.1002/j.0022-0337.2013.77.10.tb05609.x. 24098039

[6] İ. D. Çimen , T. M. Alvur , B. Coşkun , and N. E. Ö. Şükür , “Mental Health of Turkish Medical Students During the COVID‐19 Pandemic,” International Journal of Social Psychiatry 68, no. 6 (2022): 1253–1262, 10.1177/00207640211066734. 34961373

[7] D. Nugraha , S. Salamah , K. Luke , et al., “Evaluation of Health‐Related Quality of Life and Mental Health in 729 Medical Students in Indonesia During the COVID‐19 Pandemic,” Medical Science Monitor 29 (2023): e938892, 10.12659/MSM.938892. 36755476 PMC9926794

[8] M. Jupina , M. W. Sidle , and C. J. Rehmeyer Caudill , “Medical Student Mental Health During the COVID‐19 Pandemic,” Clinical Teacher 19, no. 5 (2022): e13518, 10.1111/tct.13518. 35909320 PMC9353278

[9] S. Ramachandran , M. Shayanfar , and M. Brondani , “Stressors and Mental Health Impacts of COVID‐19 in Dental Students: A Scoping Review,” Journal of Dental Education 87, no. 3 (2023): 326–342, 10.1002/jdd.13122. 36349431 PMC9877782

[10] J. Guse , A. S. Weegen , I. Heinen , and C. Bergelt , “Mental Burden and Perception of the Study Situation Among Undergraduate Students During the COVID‐19 Pandemic: A Cross‐Sectional Study and Comparison of Dental and Medical Students,” BMJ Open 11, no. 12 (2021): e054728, 10.1136/bmjopen-2021-054728. PMC8637311 34853110

[11] M. Mulyadi , S. I. Tonapa , S. Luneto , W. T. Lin , and B. O. Lee , “Prevalence of Mental Health Problems and Sleep Disturbances in Nursing Students During the COVID‐19 Pandemic: A Systematic Review and Meta‐Analysis,” Nurse Education in Practice 57 (2021): 103228, 10.1016/j.nepr.2021.103228. 34653783 PMC8496961

[12] A. Patelarou , E. A. Mechili , P. Galanis , et al., “Nursing Students, Mental Health Status During COVID‐19 Quarantine: Evidence From Three European Countries,” Journal of Mental Health 30, no. 2 (2021): 164–169, 10.1080/09638237.2021.1875420. 33504241

[13] K. Ali , E. S. A. Alhaija , M. Raja , et al., “Blended Learning in Undergraduate Dental Education: A Global Pilot Study,” Medical Education Online 28, no. 1 (2023): 2171700, 10.1080/10872981.2023.2171700. 36751853 PMC9930845

[14] I. McKerrow , P. A. Carney , H. Caretta‐Weyer , M. Furnari , and A. Miller Juve , “Trends in Medical Students' Stress, Physical, and Emotional Health Throughout Training,” Medical Education Online 25, no. 1 (2020): 1709278, 10.1080/10872981.2019.1709278. 31902315 PMC6968533

[15] E. Brennan‐Wydra , H. W. Chung , N. Angoff , et al., “Maladaptive Perfectionism, Impostor Phenomenon, and Suicidal Ideation Among Medical Students,” Academic Psychiatry 45, no. 6 (2021): 708–715, 10.1007/s40596-021-01503-1. 34350548

[16] L. N. Dyrbye , M. R. Thomas , and T. D. Shanafelt , “Systematic Review of Depression, Anxiety, and Other Indicators of Psychological Distress Among U.S. and Canadian Medical Students,” Academic Medicine 81, no. 4 (2006): 354–373, 10.1097/00001888-200604000-00009. 16565188

[17] J. Hu , J. Wang , D. Li , et al., “Mediating Effect of Sleep Disorder Between Low Mental Health Literacy and Depressive Symptoms Among Medical Students: The Roles of Gender and Grade,” Frontiers in Psychiatry 13 (2022): 818295, 10.3389/fpsyt.2022.818295. 35185657 PMC8855242

[18] W. Zeng , R. Chen , X. Wang , Q. Zhang , and W. Deng , “Prevalence of Mental Health Problems Among Medical Students in China: A Meta‐Analysis,” Medicine 98, no. 18 (2019): e15337, 10.1097/MD.0000000000015337. 31045774 PMC6504335

[19] W. I. Chan , P. Batterham , H. Christensen , and C. Galletly , “Suicide Literacy, Suicide Stigma and Help‐Seeking Intentions in Australian Medical Students,” Australasian Psychiatry 22, no. 2 (2014): 132–139, 10.1177/1039856214522528. 24526795

[20] O. S. Alhothali , A. K. Bahakim , S. M. Alharthi , et al., “Prevalence and Associated Factors of Suicidal Ideation and Attempts Among Undergraduate Medical Students of Umm Al‐Qura University, Saudi Arabia: A Cross Sectional Study,” Medical Science 25, no. 115 (2021): 2213–2221.

[21] World Health Organization , Supporting the Mental Health and Well‐Being of the Health and Care Workforce (World Health Organization, 2021).

[22] I. Qureshi , J. Chaloner , M. Gogoi , et al., “Caring for Those Who Take Care of Others: Developing Systemic and Sustainable Mental Health Support for the Diverse Healthcare Workforce in the United Kingdom,” International Journal of Environmental Research and Public Health 20, no. 4 (2023): 3242, 10.3390/ijerph20043242. 36833937 PMC9964273

[23] C. E. Hall , J. Milward , C. Spoiala , et al., “The Mental Health of Staff Working on Intensive Care Units Over the COVID‐19 Winter Surge of 2020 in England: A Cross Sectional Survey,” British Journal of Anaesthesia 128, no. 6 (2022): 971–979, 10.1016/j.bja.2022.03.016. 35465953 PMC8942706

[24] World Population Review , “World Population by Country 2024 (Live),” accessed July 6, 2024, https://worldpopulationreview.com/.

[25] M. Ur Rehman , A. Ahmed , A. Khan , and A. Khan , “The Role of Poverty, Food Security, and Rapid Population Growth on Human Development in Pakistan,” NUST Journal of Social Sciences and Humanities 8, no. 3 (2023): 89–105, 10.51732/njssh.v8i3.155.

[26] Pakistan Medical and Dental Council , “Pakistan Medical and Dental Council—Institutions & Qualifications,” accessed December 25, 2024, https://pmdc.pk/colleges.

[27] F. Chishti , H. Hassan , and S. R. Qazi , “Dental Anxiety Among Students of Lahore, Pakistan,” Pakistan Journal of Medical and Health Sciences 15, no. 9 (2021): 2659–2661, 10.53350/pjmhs211592659.

[28] F. Faul , E. Erdfelder , A. Buchner , and A.‐G. Lang , “Statistical Power Analyses Using G* Power 3.1: Tests for Correlation and Regression Analyses,” Behavior Research Methods 41, no. 4 (2009): 1149–1160. 19897823 10.3758/BRM.41.4.1149

[29] K. Kroenke , R. L. Spitzer , and J. B. W. Williams , “The PHQ‐9: Validity of a Brief Depression Severity Measure,” Journal of General Internal Medicine 16, no. 9 (2001): 606–613, 10.1046/j.1525-1497.2001.016009606.x. 11556941 PMC1495268

[30] J. Lee , E. H. Lee , and S. H. Moon , “Systematic Review of the Measurement Properties of the Depression Anxiety Stress Scales–21 by Applying Updated COSMIN Methodology,” Quality of Life Research 28, no. 9 (2019): 2325–2339, 10.1007/s11136-019-02177-x. 30937732

[31] The R Project , “R: The R Project for Statistical Computing,” accessed June 30, 2023, https://www.r‐project.org.

[32] Y. Sun , Z. Kong , Y. Song , J. Liu , and X. Wang , “The Validity and Reliability of the PHQ‐9 on Screening of Depression in Neurology: A Cross Sectional Study,” BMC Psychiatry 22, no. 1 (2022): 98, 10.1186/s12888-021-03661-w. 35139810 PMC8827244

[33] P. S. Indu , T. V. Anilkumar , K. Vijayakumar , et al., “Reliability and Validity of PHQ‐9 When Administered by Health Workers for Depression Screening Among Women in Primary Care,” Asian Journal of Psychiatry 37 (2018): 10–14, 10.1016/j.ajp.2018.07.021. 30096447

[34] C. Zanon , R. E. Brenner , M. N. Baptista , et al., “Examining the Dimensionality, Reliability, and Invariance of the Depression, Anxiety, and Stress Scale–21 (DASS‐21) Across Eight Countries,” Assessment 28, no. 6 (2021): 1531–1544, 10.1177/1073191119887449. 31916468

[35] W. S. W. Husain , A. Othman , N. A. N. Othman , W. N. W. Mohamad , and M. N. Zakaria , “Determining the Internal and External Reliability of Depression, Anxiety and Stress Scales (DASS‐21) in Assessing Psychological Symptoms Among Patients With Tinnitus,” NeuroQuantology 16, no. 12 (2018): 67–72, 10.14704/nq.2018.16.12.1876.

[36] Z. Jowkar , M. Masoumi , and H. Mahmoodian , “Psychological Stress and Stressors Among Clinical Dental Students at Shiraz School of Dentistry, Iran,” Advances in Medical Education and Practice 11 (2020): 113–120, 10.2147/AMEP.S236758. 32104133 PMC7024806

[37] R. Tripathi , S. S. Alqahtani , A. M. Meraya , H. A. Makeen , P. Tripathi , and S. S. Pancholi , “Evaluation of Depression, Anxiety, and Stress Among University Healthcare Students,” Journal of Applied Pharmaceutical Science 12, no. 10 (2022): 078–087, 10.7324/JAPS.2022.121008.

[38] R. Moore , L. V. Madsen , and M. Trans , “Stress Sensitivity and Signs of Anxiety or Depression Among First Year Clinical Dental and Medical Students,” Open Journal of Medical Psychology 9, no. 1 (2020): 7–20, 10.4236/ojmp.2020.91002.

[39] A. R. Lerman , K. K. Yamamoto , G. W. Taylor , and S. G. Saeed , “High Depressive Symptom Prevalence in Dental Students Associated With Lifestyle and Well‐Being Characteristics,” Journal of Dental Education 84, no. 7 (2020): 771–780, 10.1002/jdd.12144. 32216145

[40] M. Á. de Oliveira Viana , É. Porto , L. dos Santos Dantas , F. D. Soares Forte , S. D'Ávila Lins Bezerra Cavalcanti , and A. C. de Lima Targino Massoni , “Depression, Anxiety, and Stress in Dental Students and Associations with Sociodemographic Variables and the Academic Environment,” Trends in Psychology (2024): 1–9, 10.1007/s43076-024-00359-2.

[41] M. D. Finkelman , A. Joseph , S. Khoynezhad , and T. B. Bordin , “Depressive Symptoms and Their Correlates Among Predoctoral Dental Students in the United States,” Journal of Dental Education 88, no. 6 (2024): 856–864, 10.1002/jdd.13492. 38348972

[42] S. Moradi , M. S. Fateh , E. Movahed , et al., “The Prevalence of Depression, Anxiety, and Sleep Disorder Among Dental Students: A Systematic Review and Meta‐Analysis,” Journal of Dental Education 88, no. 7 (2024): 900–909, 10.1002/jdd.13506. 38504501

[43] W. Husain , “Barriers in Seeking Psychological Help: Public Perception in Pakistan,” Community Mental Health Journal 56, no. 1 (2020): 75–78, 10.1007/s10597-019-00464-y. 31542848

[44] F. R. Choudhry , N. Khan , and K. Munawar , “Barriers and Facilitators to Mental Health Care: A Systematic Review in Pakistan,” International Journal of Mental Health 52, no. 2 (2023): 124–162, 10.1080/00207411.2021.1941563.

[45] N. Imran , I. I. Haider , M. R. Bhatti , A. Sohail , and M. Zafar , “Prevalence of Psychoactive Drug Use Among Medical Students in Lahore,” Annals of King Edward Medical University 17, no. 4 (2011): 343–346.

[46] S. Zada , Y. Wang , M. Zada , and F. Gul , “Effect of Mental Health Problems on Academic Performance Among University Students in Pakistan,” International Journal of Mental Health Promotion 23, no. 3 (2021): 395–408, 10.32604/IJMHP.2021.015903.

[47] A. W. Yousafzai , S. Ahmer , E. Syed , et al., “Well‐Being of Medical Students and Their Awareness on Substance Misuse: A Cross‐Sectional Survey in Pakistan,” Annals of General Psychiatry 8 (2009): 8, 10.1186/1744-859X-8-8. 19228374 PMC2660326

[48] J. Yates , “Development of a “Toolkit” to Identify Medical Students at Risk of Failure to Thrive on the Course: An Exploratory Retrospective Case Study,” BMC Medical Education 11, no. 1 (2011): 95, 10.1186/1472-6920-11-95. 22098629 PMC3229499

[49] S. Clement , O. Schauman , T. Graham , et al., “Mental Health Stigma and Access to Care: A Systematic Review of Quantitative and Qualitative Studies,” Psychological Medicine 45, no. 1 (2014): 11–27. 24569086 10.1017/S0033291714000129

[50] S. Clement , O. Schauman , T. Graham , et al., “What Is the Impact of Mental Health‐Related Stigma on Help‐Seeking? A Systematic Review of Quantitative and Qualitative Studies,” Psychological Medicine 45, no. 1 (2015): 11–27, 10.1017/S0033291714000129. 24569086

[51] B. E. Vaa Stelling and C. P. West , “Faculty Disclosure of Personal Mental Health History and Resident Physician Perceptions of Stigma Surrounding Mental Illness,” Academic Medicine 96, no. 5 (2021): 682–685, 10.1097/ACM.0000000000003941. 33496429

[52] S. Clement , O. Schauman , T. Graham , et al., “What Is the Impact of Mental Health‐Related Stigma on Help‐Seeking?,” Psychological Medicine 45, no. 1 (2015): 11–27. 24569086 10.1017/S0033291714000129

[53] A. Gulliver , L. Farrer , K. Bennett , et al., “University Staff Experiences of Students With Mental Health Problems and Their Perceptions of Staff Training Needs,” Journal of Mental Health 27, no. 3 (2018): 247–256, 10.1080/09638237.2018.1466042. 29722579

[54] R. Delderfield , M. Ndoma‐Egba , K. Riches‐Suman , and J. Boyne , “A Learning Development‐Faculty Collaborative Exploration of Postgraduate Research Student Mental Health in a UK University,” Journal of Learning Development in Higher Education 18 (2020): 1–30, 10.47408/jldhe.vi18.571.

[55] E. Sheldon , M. Simmonds‐Buckley , C. Bone , et al., “Prevalence and Risk Factors for Mental Health Problems in University Undergraduate Students: A Systematic Review With Meta‐Analysis,” Journal of Affective Disorders 287 (2021): 282–292, 10.1016/j.jad.2021.03.054. 33812241

[56] M. R. Hill , S. Goicochea , and L. J. Merlo , “In Their Own Words: Stressors Facing Medical Students in the Millennial Generation,” Medical Education Online 23, no. 1 (2018): 1530558, 10.1080/10872981.2018.1530558. 30286698 PMC6179084

[57] F. Nadir , H. Sardar , and H. Ahmad , “Perceptions of Medical Students Regarding Brain Drain and Its Effects on Pakistan's Socio‐Medical Conditions: A Cross‐Sectional Study,” Pakistan Journal of Medical Sciences 39, no. 2 (2023): 401–403, 10.12669/pjms.39.2.7139. 36950443 PMC10025733

[58] Z. Tariq , A. Aimen , U. Ijaz , and K. U. R. Khalil , “Career Intentions and Their Influencing Factors Among Medical Students and Graduates in Peshawar, Pakistan: A Cross‐Sectional Study on Brain Drain,” Cureus 15, no. 11 (2023): e48445. 38074031 10.7759/cureus.48445 PMC10702615

[59] C. Thorley , Not by Degrees Improving Student Mental Health in the UK'S Universities (IPPR, 2017).

[60] K. Ali , C. Tredwin , E. J. Kay , A. Slade , and J. Pooler , “Preparedness of Dental Graduates for Foundation Training: A Qualitative Study,” British Dental Journal 217, no. 3 (2014): 145–149, 10.1038/sj.bdj.2014.648. 25104700

[61] Z. Mat Yudin , K. Ali , W. M. A. Wan Ahmad , et al., “Self‐Perceived Preparedness of Undergraduate Dental Students in Dental Public Universities in Malaysia: A National Study,” European Journal of Dental Education 24, no. 1 (2020): 163–168, 10.1111/eje.12480. 31698535

[62] K. Ali , E. McColl , C. Tredwin , S. Hanks , C. Coelho , and R. Witton , “Addressing Racial Inequalities in Dental Education: Decolonising the Dental Curricula,” British Dental Journal 230, no. 3 (2021): 165–169, 10.1038/s41415-020-2598-z. 33574542 PMC7877507

[63] M. O'Reilly , “Social Media and Adolescent Mental Health: The Good, the Bad and the Ugly,” Journal of Mental Health 29, no. 2 (2020): 200–206, 10.1080/09638237.2020.1714007. 31989847

[64] P. M. Valkenburg , “Social Media Use and Well‐Being: What We Know and What We Need to Know,” Current Opinion in Psychology 45 (2022): 101294, 10.1016/j.copsyc.2021.12.006. 35016087

[65] A. I. M. B. Al‐Moadhadi , R. M. A. M. Al‐Naema , and K. Ali , “InstaPower Opportunities for Dental Students to Connect With Patients Without Borders,” European Journal of Dental Education 27, no. 3 (2023): 763–764, 10.1111/eje.12864. 36315440

