# Supplementary material for: Beyond the Smile: Exploring the Mental Well‐Being of Dental Students Across Institutions
Source: Eur J Dent Educ. 2025 Feb 24;29(2):451–61. doi: 10.1111/eje.13085 (PMC12006705; doi:10.1111/eje.13085)
Supplement: Supplementary file 1 — Appendix S1 [file EJE-29-451-s001.docx]

Evaluation of the Mental Health of Undergraduate Dental Students in Pakistan

This study is being undertaken by a group of Dental academics to evaluate the frequency and patterns of mental health issues amongst undergraduate dental students in Pakistan.

* Indicates required question

**Informed consent**

Your participation is completely voluntary. You are requested to conﬁrm all statements below.

# I am over 18 years old *

*Mark only one option*


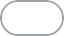
 Yes

# I understand that my involvement in this study and particular data * from this research will remain strictly conﬁdential. Only researchers involved in the study will have access to the data.

*Mark only one option*


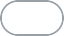
 Yes

# I fully and freely consent to participate in the study *

*Mark only one option*


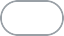
 Yes
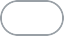
 No

**Demographics**

# Age (Years) *

*Mark only one option*


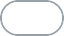
 18 -21


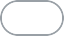
 22-25


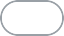
 26-29


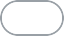
 30 or more

# Gender *

*Mark only one option*

Male Female Other:

# Institution Name and City

1. Institution Type *

*Mark only one option*


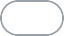
 Public
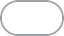
 Private
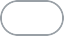
 Other

# Year of Study *

*Mark only one option*


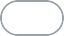
 Year 1


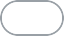
 Year 2


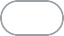
 Year 3


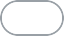
 Year 4


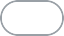
 Year 5


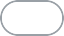
 Year 6

# Financial support

*Mark only one option*


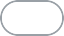
 Self-ﬁnance
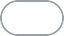
 Sponsorship
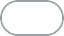
 Scholarship
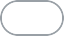
 Other

**Section B**

**Over the last 2 weeks**, how often have you been bothered by any of the following problems? Please read and answer each of the following statements.

The rating scale is as follows: Not at all

Several days

More than half the days Nearly every day

# Little interest or pleasure in doing things

*Mark only one option*


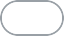
 Not at all
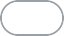
 Several days


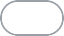
 More than half the days
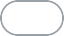
 Nearly every day

# Feeling down, depressed, or hopeless

*Mark only one option*


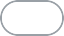
 Not at all
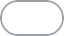
 Several days


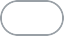
 More than half the days
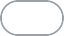
 Nearly every day

# Trouble falling or staying asleep, or sleeping too much

*Mark only one option*


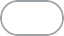
 Not at all
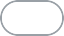
 Several days


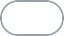
 More than half the days
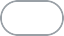
 Nearly every day

# Feeling tired or having little energy

*Mark only one option*


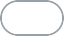
 Not at all
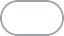
 Several days


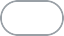
 More than half the days
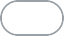
 Nearly every day

# Poor appetite or overeating

*Mark only one option*


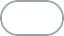
 Not at all
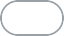
 Several days


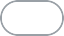
 More than half the days
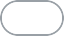
 Nearly every day

# Feeling bad about yourself — or that you are a failure or have let yourself or your family down

*Mark only one option*


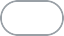
 Not at all
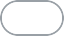
 Several days


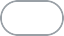
 More than half the days
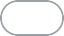
 Nearly every day

# Trouble concentrating on things, such as reading the newspaper or watching television

*Mark only one option*


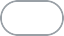
 Not at all
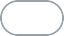
 Several days


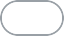
 More than half the days
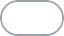
 Nearly every day

# Moving or speaking so slowly that other people could have

noticed? Or the opposite — being so ﬁdgety or restless that you have been moving around a lot more than usual

*Mark only one option*


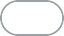
 Not at all
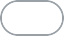
 Several days


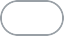
 More than half the days
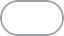
 Nearly every day

# Thoughts that you would be better off dead or of hurting yourself in some way

*Mark only one option*


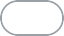
 Not at all
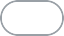
 Several days


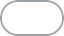
 More than half the days
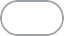
 Nearly every day

**Section C**

Please read and answer each of the following statements indicating how much the statement applied to you **over the past week.** There are no right or wrong answers. Do not spend too much time on any statement.

The rating scale is as follows:

Did not apply to me at all

Applied to me to some degree, or some of the time

Applied to me to a considerable degree or a good part of time Applied to me very much or most of the time

# I found it hard to wind down

*Mark only one option*


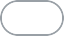
 Did not apply to me at all


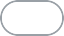
 Applied to me to some degree, or some of the time


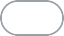
 Applied to me to a considerable degree or a good part of time
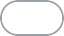
 Applied to me very much or most of the time

# I was aware of dryness of my mouth

*Mark only one option*


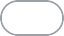
 Did not apply to me at all


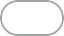
 Applied to me to some degree, or some of the time


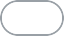
 Applied to me to a considerable degree or a good part of time
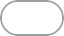
 Applied to me very much or most of the time

# I could not seem to experience any positive feeling at all

*Mark only one option*


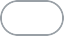
 Did not apply to me at all


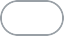
 Applied to me to some degree, or some of the time


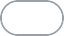
 Applied to me to a considerable degree or a good part of time
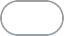
 Applied to me very much or most of the time

# I experienced breathing diﬃculty (e.g. excessively rapid breathing, breathlessness in the absence of physical exertion)

*Mark only one option*


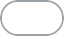
 Did not apply to me at all


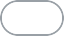
 Applied to me to some degree, or some of the time


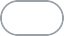
 Applied to me to a considerable degree or a good part of time
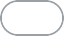
 Applied to me very much or most of the time

# I found it diﬃcult to work up the initiative to do things

*Mark only one option*


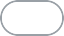
 Did not apply to me at all


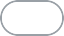
 Applied to me to some degree, or some of the time


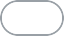
 Applied to me to a considerable degree or a good part of time
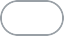
 Applied to me very much or most of the time

# I tended to over-react to situations

*Mark only one option*


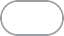
 Did not apply to me at all


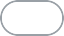
 Applied to me to some degree, or some of the time


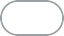
 Applied to me to a considerable degree or a good part of time
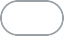
 Applied to me very much or most of the time

# I experienced trembling (e.g. in the hands)

*Mark only one option*


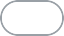
 Did not apply to me at all


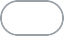
 Applied to me to some degree, or some of the time


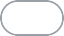
 Applied to me to a considerable degree or a good part of time
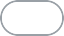
 Applied to me very much or most of the time

# I felt that I was using a lot of nervous energy

*Mark only one option*


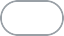
 Did not apply to me at all


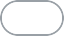
 Applied to me to some degree, or some of the time


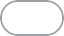
 Applied to me to a considerable degree or a good part of time
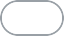
 Applied to me very much or most of the time

# I was worried about situations in which I might panic and make a fool of myself

*Mark only one option*


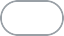
 Did not apply to me at all


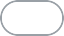
 Applied to me to some degree, or some of the time


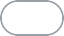
 Applied to me to a considerable degree or a good part of time
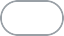
 Applied to me very much or most of the time

# I felt that I had nothing to look forward to

*Mark only one option*


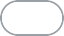
 Did not apply to me at all


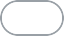
 Applied to me to some degree, or some of the time


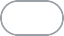
 Applied to me to a considerable degree or a good part of time
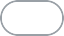
 Applied to me very much or most of the time

# I found myself getting agitated

*Mark only one option*


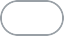
 Did not apply to me at all


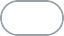
 Applied to me to some degree, or some of the time


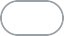
 Applied to me to a considerable degree or a good part of time Applied to me very much or most of the time

# I found it diﬃcult to relax

*Mark only one option*

Did not apply to me at all

Applied to me to some degree, or some of the time

Applied to me to a considerable degree or a good part of time Applied to me very much or most of the time

# I felt down-hearted and blue

*Mark only one option*

Did not apply to me at all

Applied to me to some degree, or some of the time

Applied to me to a considerable degree or a good part of time Applied to me very much or most of the time

# I was intolerant of anything that kept me from getting on with what I was doing

*Mark only one option*

Did not apply to me at all

Applied to me to some degree, or some of the time

Applied to me to a considerable degree or a good part of time Applied to me very much or most of the time

# I felt I was close to panic

*Mark only one option*

Did not apply to me at all

Applied to me to some degree, or some of the time

Applied to me to a considerable degree or a good part of time Applied to me very much or most of the time

# I was unable to become enthusiastic about anything

*Mark only one option*

Did not apply to me at all

Applied to me to some degree, or some of the time

Applied to me to a considerable degree or a good part of time Applied to me very much or most of the time

# I felt I was not worth much as a person

*Mark only one option*

Did not apply to me at all

Applied to me to some degree, or some of the time

Applied to me to a considerable degree or a good part of time Applied to me very much or most of the time

# I felt that I was rather touchy

*Mark only one option*

Did not apply to me at all

Applied to me to some degree, or some of the time

Applied to me to a considerable degree or a good part of time Applied to me very much or most of the time

# I was aware of the action of my heart in the absence of physical exertion (e.g. sense of heart rate increase, heart missing a beat)

*Mark only one option*

Did not apply to me at all

Applied to me to some degree, or some of the time

Applied to me to a considerable degree or a good part of time Applied to me very much or most of the time

# I felt scared without any good reason

*Mark only one option*

Did not apply to me at all

Applied to me to some degree, or some of the time

Applied to me to a considerable degree or a good part of time Applied to me very much or most of the time

# I felt that life was meaningless

*Mark only one option*

Did not apply to me at all

Applied to me to some degree, or some of the time

Applied to me to a considerable degree or a good part of time Applied to me very much or most of the time

If you believe you are experiencing mental health issues please answer the questions below:

1. In your opinion, what are the key factors affecting your mental health adversely?
2. How could your institution support you better to manage your mental health?

Thank you for your participation!

This content is neither created nor endorsed by Google.

[Forms](https://www.google.com/forms/about/?utm_source=product&utm_medium=forms_logo&utm_campaign=forms)
